# Dementia Caregiver Virtual Support—An Implementation Evaluation of Two Pragmatic Models during COVID-19

**DOI:** 10.3390/geriatrics6030080

**Published:** 2021-08-19

**Authors:** Jacy A. Weems, Shana Rhodes, James S. Powers

**Affiliations:** 1 Department of Health Policy, Vanderbilt University School of Medicine, Nashville, TN 37212, USA; jacy.a.weems@vanderbilt.edu; 2 Center for Quality Aging, Vanderbilt University School of Medicine, Nashville, TN 37212, USA; shana.atkins@vumc.org; 3 Tennessee Valley Healthcare System Geriatric Research Education and Clinical Center, Nashville, TN 37232, USA

**Keywords:** caregiver support, telehealth, dementia care, COVID-19

## Abstract

Caregivers of people with Alzheimer’s and related dementias (ADRD) require support. Organizations have pivoted from traditional in-person support groups to virtual care in the face of the COVID-19 pandemic. We describe two model programs and their pragmatic implementation of virtual care platforms for ADRD caregiver support. A mixed methods analysis of quantitative outcomes as well as a thematic analysis from semi-structured interviews of facilitators was performed as part of a pragmatic quality improvement project to enhance delivery of virtual support services for ADRD caregivers. Implementation differed among individual organizations but was well received by facilitators and caregivers. While virtual platforms can present challenges, older adults appreciated the strength of group facilitators and reported enhanced connectedness related to virtual support. Barriers to success include the limitations of virtual programming, including technological issues and distractions from program delivery. Virtual support can extend outreach, addressing access and providing safe care during a pandemic. Implementation differs among organizations; however, some elements of virtual support may be long-lasting.

## 1. Introduction

Caregivers of people with Alzheimer’s and related dementias (ADRD) are at a greater risk for anxiety, depression and poorer quality of life. The COVID-19 pandemic and associated physical distancing measures increased the loneliness and social isolation of both persons living with ADRD and their caregivers due to fewer caregiver opportunities for in-person coping strategies, socialization, support and respite [1,2]. The global pandemic also had notable consequences on home and community-based services, including caregiver support services. Caregiver support programs across all sectors pivoted quickly from in-person to virtual care to address prevalent needs and continue engagement [2]. We describe how two organizations (a VA medical center and a statewide non-profit) collaborating within the Middle Tennessee Geriatric Workforce Enhancement Program [3] implemented differing virtual caregiver support platforms during the COVID-19 pandemic.

### Context

Caregivers First is a structured program developed by the Department of Veterans Affairs (VA) to support caregivers of cognitively and/or functionally impaired community-dwelling veterans. The goal of the program is to strengthen caregivers and veterans’ access to VA services, reduce caregiver feelings of isolation, and increase veteran days in the community [4]. The Tennessee Valley Healthcare System (TVHS), a regional integrated healthcare system, is comprised of two medical centers and twelve community-based outpatient clinics serving over 100,000 patients annually. Caregivers First facilitators and other program staff were primarily based out of the TVHS Nashville VA Medical Center campus. Outpatient clinics throughout the TVHS system typically provide referrals to the Caregivers First program. Once enrolled, participants voluntarily attend four sessions with trained facilitators once per week for an hour. In-person support groups were piloted in 2019 at TVHS and expanded to include telephone and video sessions prior to the COVID-19 pandemic. Implementation of virtual Caregivers First sessions was meant to increase program access for rural caregivers. Facilitators led four group support cohorts prior to COVID-19 restrictions serving 22 caregivers. Another six caregivers were provided four group support classes during the pandemic as a continuation of the program.

Alzheimer’s Tennessee, a non-profit organization established in 1983, provides caregiver education and direct support throughout the state to families impacted by a dementia diagnosis. Available services include a number of programs, such as the agency’s locally staffed helpline, training workshops, help with local resources and referrals for caregivers in crisis and more than 42 in-person social support groups throughout the state. Referrals were primarily made by identifying caregivers in crisis on the statewide hotline; however, caregiver support groups provided another opportunity to identify distressed caregivers who were then offered a referral to local Alzheimer’s Tennessee chapters for resources and service enrollment. In 2020, Alzheimer’s Tennessee implemented a twice-weekly open access virtual caregiver support group with statewide outreach. Caregivers were invited to attend support groups voluntarily at any time.

## 2. Materials and Methods

### 2.1. Study Methods

We designed the mixed-methods study to meet Standards for QUality Improvement Reporting Excellence (SQUIRE) criteria and the Quality Improvement Minimum Quality Criteria Set (QI-MQCS) domains for appraising quality improvement work [5,6]. 

### 2.2. Quantitative Data

Caregivers First demographic information was gathered at the first session. Burden and depression scores were self-assessed at each of the first and last class sessions using the 4-item Zarit Burden Interview Scale [7] and the PHQ-2 depression questionnaire [8]. The short 4-item Zarit Burden Interview scale is ranked on a 5-point Likert scale for a possible maximum score of 16. PHQ-2 scores range from 0 to 6; scores greater than or equal to 3 are a positive indication for depression. Each of these values were self-assessed and gathered using a written form included in the participant workbook; facilitators gathered and reported this information. We calculated travel time and distance saved by each caregiver participating by inputting each caregiver’s primary address and the Nashville Campus address into Google Maps. 

Facilitator role was gathered for comparison of program satisfaction and implementation outcomes. Facilitators of Caregivers First sessions were of varied disciplines, including physicians, social workers, pharmacists, and nurse practitioners. Facilitators who were identified to be interviewed were both social workers working in the VA Caregiver Support Program.

Alzheimer’s Tennessee did not implement pre- or post-session surveys for caregivers but did share programmatic information including the rate of caregivers referred to local Alzheimer’s Tennessee chapters for localized resources and enrollment in services. Participants educated on virtual platforms and session frequency were also shared. Alzheimer’s Tennessee facilitators included trained program specialists with strong backgrounds in dementia care and caregiver support.

### 2.3. Qualitative Data

Our study design was informed by the Consolidated Framework for Implementation Research (CFIR) [9] to evaluate the implementation of two virtual caregiver support programs implemented within the Middle Tennessee Geriatrics Workforce Enhancement Program (GWEP) [3]. Semi-structured interviews were conducted with two facilitators from each program by J.A.W. Expert sampling was used to identify facilitators within both Caregivers First and Alzheimer’s Tennessee; this rendered high-yield interviews under limited time constraints. Each participant provided verbal consent, and all collected data were de-identified for the purposes of our study. 

Themes were categorized within outlined domains within the CFIR evaluation framework: inner setting, or specific organizational context; outer setting, or the broader social context; individuals involved; process; and intervention. Codes were generated using a template analysis; inclusion and exclusion criteria for domains and constructs were defined within the CFIR Codebook Template which as adapted and used for analysis [9]. Thematic concordance between authors J.A.W. and J.S.P. was greater than 80%. General caregiver comments on program satisfaction for Caregivers First were captured in mailed open-ended post-surveys to drive future quality improvement opportunities.

## 3. Results

### 3.1. Caregivers First

Caregivers First participants (2019–2021) included 28 unique individuals (Table 1). Attendance mode included: 2 in-person (7%), 9 video (32%), and 17 telephone (61%). An average of 2.5 caregivers attended each class session with two to six participants in each class. A total of four caregiver support educational series (16 class sessions) were held between 2019 and 2021. 

Participating caregivers saved a compiled 8640 miles and 172 hours of drive time. Caregivers lived an average of 44 miles away from the medical center. Veterans being cared for had varied conditions. Most participants were caring for a veteran with an ADRD diagnosis (*n* = 25), while two cared for someone living with multiple sclerosis and one with Parkinson’s disease. Participant characteristics can be seen in Table 1.

In addition to technical requirements for distance engagement, staff committed two and a half hours per class to prepare, facilitate, document and provide referrals and post-course support services for identified veteran or caregiver needs. The facilitators’ successful recruitment efforts addressed participation hesitancy through reminders and discussing reservations one-on-one. However, this was often the most time-consuming preparatory task.

Themes identified by the semi-structured interviews of facilitators are included in Table 2.

### 3.2. Facilitator Themes

The Process domain, particularly within the Engaging construct, was inferred primarily as a strength for facilitators, as the structured and comprehensive content included in the facilitator training, facilitator handbook and participant workbook empowered facilitators in content delivery. Individuals involved, including the skilled social workers facilitating this program, contributed to participants and facilitators’ overall program satisfaction.

The potential improvement areas participants highlighted included: 1. Outer setting—a desire for a bigger group of participating caregivers to stimulate conversation; 2. Inner setting—most caregivers suggested additional educational sessions offered on a less-frequent basis (once a month instead of weekly) as caregivers are overburdened with daily caregiving responsibilities; and 3. Intervention—some participants joining in-person had difficulty hearing those joining by phone, and they suggested technological improvements for those participating. 

### 3.3. Participant Feedback

Mailed post-course surveys were completed by 12 (43%) caregivers and allowed our study team to gather information on program satisfaction and initial program outputs. Caregivers’ post-program testimonials noted feeling “encourage[d] to be a better caregiver” and “helped me to know I was not the only one that needed a break or just to get outside and enjoy nature for a while to relax”. Within the Process domain, program strengths included the comprehensive participant workbook and relatable session topics. Technical challenges were identified by both facilitators and participants.

### 3.4. Alzheimer’s Tennessee

Alzheimer’s Tennessee implemented and expanded support group offerings online, with four weekly support groups, each hosting caregivers statewide. Support groups were led by regional staff members, but participants could attend any group, regardless of their residence. Three-part topical caregiver education programs were held in each of the major regions of the state (East, West, and Middle Tennessee). Alzheimer’s Tennessee decided to host monthly educational webinars driven by caregiver-selected topics. 

Four live caregiver support sessions are open throughout the week to all Tennessee caregivers or those caring for loved ones living in Tennessee. An average of 15 participants attended each session with a referral rate of 0.2 to local Alzheimer’s Tennessee chapter representatives and resources. In addition, nine topical educational sessions were held in each region of the state (East, Middle, and West) to complement the series 

Themes identified by semi-structured interviews of Alzheimer’s Tennessee support group facilitators are included in Table 3. Virtual Alzheimer’s Tennessee support group facilitators were primarily selected based on experience in coordinating other initiatives supporting caregivers of those living with dementia. The sessions were loosely structured to allow for caregivers to primarily drive discussion based on real-time issues they were experiencing. Referral information and other programmatic data were tracked, collected and shared by Alzheimer’s Tennessee through internal mechanisms. No direct feedback was obtained from participants regarding program satisfaction and implementation.

### 3.5. Facilitator Feedback

Feedback from facilitators focused on positive aspects within the following domains: 1. Outer setting—accessibility of virtual support; 2. Inner setting—adaptability permitting caregivers minimized time away from loved ones to participate in the virtual support groups; and 3. Individuals involved—virtual support utilized strong organizational resources and knowledge of many dementia professionals.

Potential improvement areas that facilitators highlighted included: 1. Process—technical difficulties and lack of initial facility with the platform, and 2. Intervention—preference for in-person support meetings for enhanced communication, ability to observe nonverbal body language and a more structured group process.

## 4. Discussion

Quantitative and qualitative data were gathered through this study design. For each predominant CFIR construct, corresponding implementation facilitators and barriers were identified for each program. In Caregivers First, the domains Individuals involved and Process were seen as strengths, while elements of Outer setting, Inner setting and Intervention were potential barriers to implementation. Facilitators for Alzheimer’s Tennessee, on the other hand, identified constructs within the Outer setting, Inner setting and Individuals involved domains as strengths, while elements of the Process and Intervention domains were seen as potential barriers to implementation.

In Caregivers First, caregivers and facilitators valued the connectedness and support as well as the structured progression offered by the groups. Small group size, frequency (weekly sessions), and technical platform challenges are seen as barriers. For Alzheimer’s Tennessee, facilitators appreciated the benefits of the virtual platform in providing caregiver access and facilitating the contributions of other healthcare professionals. Facilitators preferred in-person meetings and appreciated the technical platform difficulties experienced by some caregivers. For both programs, Individuals involved remained a positive construct, and Intervention was a barrier. Both Alzheimer’s Tennessee and Caregivers First facilitators were well-versed in facilitating support groups and working with families affected by a dementia diagnosis. However, the difficulties experienced by both program participant populations in implementing virtual platforms are consistent with known challenges that older adults experience in adapting to new technology [10]. The VA Social Work Service has received resources to add Caregiver Support Coordinator personnel to sustain the program and facilitate virtual support groups moving forward. Alzheimer’s Tennessee received funding to develop staff positions overseeing virtual support services early during the pandemic and plan to keep these personnel for program sustainability.

Both programs supported caregivers dealing predominantly with ADRD. Participants and facilitators emphasized the caring human connection that persisted despite the virtual platform limitations. One facilitator feared the switch from in-person to virtual support groups as less effective and engaging, but once the program had started, believed services were delivered just as effectively in a virtual format. Attendance waned on audio conference calls during the pandemic, but once video conferencing capabilities were implemented, attendance rapidly increased according to facilitators. Particularly for those exhibiting depression or distress, the ability to read body language allows enhanced communication across group members and facilitators.

Caregiver support is a complex interaction appropriate for the complexity of care needs involved in providing dementia caregiver support. A pragmatic intervention such as virtual support may necessitate adaptation due to organizational, patient and caregiver heterogeneity [11]. Support groups were dual purpose; one main concern for facilitators was identifying caregivers in crisis and providing appropriate services or referrals. Both the VA and Alzheimer’s Tennessee have a strong organizational presence throughout the state; this allowed referral to other facilities and chapters for local caregiver resources.

### Limitations

Traditional caregiver support groups are in-person, and new models of virtual care have not been tested widely for effectiveness. Caregivers First gathered specific impact metrics (depression, caregiver burden, etc.) and direct caregiver feedback for the purposes of this report, while Alzheimer’s Tennessee did not, potentially missing improvement opportunities from the participant perspective. The limited number of participants included in both qualitative and quantitative data collection precludes statistical analysis. Only two facilitators from each program were interviewed due to time constraints, limiting maximum content saturation, but common themes emerged which will drive future improvements within the Middle Tennessee GWEP’s programming. Pre- and post-COVID programmatic outcomes for Caregivers First were mixed rather than separated due to limited survey completion by caregivers post-COVID. The virtual models presented may not be applicable to other organizations offering caregiver support programs.

## 5. Conclusions

Virtual support can extend outreach, addressing access and providing safe care during a pandemic; however, implementation differs among organizations. Some elements of virtual support may be long-lasting beyond the pandemic, as they may represent efficient ways to increase access, facilitate engagement, and address isolation. Organizations providing caregiver support should strengthen partner networks with local organizations to ensure caregivers can choose from available services to meet individual needs.

## Figures and Tables

**Table 1:** Caregivers First Participant Characteristics (*n* = 28).

Educational Sessions		
	Classes (participants/class)	16 (4.0)
Caregiver relationship		
	Wife	24 (85.7%)
	Husband	1 (3.6%)
	Daughter	2 (7.1%)
	Non-family relationship	1 (3.6%)
Sex		
	Female	27 (96%)
	Male	1 (4%)
Age		
	Years	68
Delivery method		
	In-person	2 (7%)
	Video	9 (32%)
	Telephone	17 (61%)
Mileage and travel saved		
	Total miles saved	8640
	Average participant distance	44
	Total hours of travel saved	172
	Average per participant	6
Positive Caregiver Burden Score		
	Difference (first to last class)	+1
Positive Caregiver Depression Scores (3–6)		
	Difference (first to last class)	No difference

**Table 2:** Qualitative facilitator themes within CFIR domains and constructs—Caregivers First.

CFIR Domain	CFIR Construct	Theme	Subthemes	Quotes
Outer setting	Relative advantage	VA commitment to caregivers of veterans	Aligns with VA mission National Caregiver Support Program (CSP) Exacerbated needs of veteran caregivers	President Abraham Lincoln said years ago to care for those who basically care for the veterans—so we are honoring that commitment years ago that President Lincoln made by providing care to those veteran caregivers so they can continue that care for the veteran.
	External policies and incentive	National social distancing mandates	Social isolation	I think it was actually extra important having the Caregivers Support session during the COVID pandemic, because sometimes we would be the only people they would have communication with.
Inner Setting	Adaptability	Semi-structured	Relatable Effective communication tools	It’s a grab-and go curriculum and we follow that, but a lot of the times, the things in the curriculum, it will give the caregiver a chance to express themselves and so they can express and say how they relate to a particular topic –and a lot of the times, whatever we’re talking about that time, it’s exactly what the caregiver is going through. And so, they’re able to relate to those particular topics and they’re able to share with other caregivers. Then, when they start sharing with the other caregivers, they start feeling like they’re not alone
Individuals involved	Self-efficacy	Facilitators have a wealth of caregiver support experience	Personal experience Professional experience	I do have a history of doing groups, so I do hope I have the skill especially being a social worker just being openly able talk to someone and also, you know, if you do groups, they tend to kinda flow. They tend to talk to each other and listen to each other so it just kinda flows through.
Process	Engaging	Structured implementation	Facilitator training Facilitator handbook Participant workbooks	They provided a training for us, so we were able to go over even that training of those materials with them, if we had any questions, before we presented the training with the caregivers, so no, I think it went very smoothly.
	Executing	Technical issues Fluctuation in attendance	Communication Lack of piloting No-shows	Some people were further away and didn’t want to drive to Nashville Campus. They went to one of our CBOCs and the goal there was they go into the clinic and the clinics could connect them to [the] group virtually. On one instance, one person didn’t show up as scheduled to the clinic; another instance, they had technical challenges and wasn’t able to set up, or we weren’t able to hear them clearly. So, of course, we’re trying to call the clinic, get a representative to go to the group room to help the caregiver figure out what’s wrong with the computer. So, you know, you just have those little snags, and you ask for forgiveness and you move forward.
Intervention	Relative priority	Accessibility	Easy to access Barriers for those without internet access Participant distractions	Our caregivers live all over middle TN. And driving to downtown Nashville because of the distance, because of the traffic, because of the parking now they don’t have to, that barrier is alleviated when we do it virtually. Because you’re on that computer, because you don’t have that personal interaction, you may hear background noise, or the caregiver may be taking care of veteran and participate at the same time, so the caregiver is trying to multitask.
	Design quality and packaging	Empowering caregivers to identify needs	Well-structured Caregiver-centered	I—honestly I think it really went according to the, to the plan—other than having to do it by VVC—but the caregivers first started in, I think in Durham—they have a really, have this set up so well. The way we document in the records so there is a template set up. They give us a subject per session. I think the VA as well as other companies can see now that, you know, we can provide good services virtually, that we have the capability of doing that. And veterans and caregivers can still receive quality services virtually. You don’t necessarily have to be in the office or directly in-person with someone to receive good quality care.
	Reflecting and evaluating	Need to address access barriers	Technological equipment Broadband access	You know, one thing that I do feel that the veterans and caregivers would benefit and you know if we were like able to provide them with a tablet or with a laptop or something that they would be able to use for those who do not have it —if we would be able to, to issue those, because there is that gap of the people that really would like to join, but that they’re not able to. Of course, you know, we would also run into if they have the internet capability as well.

**Table 3:** Qualitative facilitator themes within CFIR domains and constructs—Alzheimer’s Tennessee.

Domain	CFIR Construct	Theme	Subthemes	Quotes
Outer Setting	Relative advantage	Outreach to greater number of caregivers	Ease of accessibility Eliminates geographic barriers	I have to say, it’s been a wonderful service to be able to give to people. And we likely will maintain some family caregiver support groups too because when you can just take an hour out of your week and just go log onto your computer and your loved one can be with you, in a manner of speaking, in the same house or the same room, the barriers are gone, and you have a place to be heard and to receive support.
	External policies and incentives	National social distancing mandates	Exacerbated needs Lack of caregiver support	Once the restrictions of the pandemic were in place, we knew we had to continue to offer support services. Just because the pandemic stopped a lot of things, or the restrictions did, Alzheimer’s didn’t stop and the need for supports and services didn’t stop—if anything it was exacerbated. So, we made a decision about a year ago at this time to go virtual as a support group.
Inner Setting	Adaptability	Contact time with caregivers Addresses barriers to social support services Easy referral for additional services	Frequency Minimize time away from loved one with dementia Area Agency on Aging Local Alzheimer’s TN Chapters	Virtually, we’re doing it every week, because we felt that people needed to be connected and they need that check-in once a week and for some, that’s all they get. Also, even one challenge can be “Who is going to take care of my loved one while I attend the support groups?” Just for an example, we have one lady who attends a support group… early on, she said “Now, I’m going to be on this support group, but I probably won’t say anything, but I just love to hear what folks are saying because that does help me. The reason I won’t say anything is because my husband is right here, but at least I can hear what is going on and I can receive support in that sense too.” If anything, it just provided another platform to get the support they receive. There was a man on the [Caregiver Support Group] line, and you could tell he was very desperate caring for his mom… we fill out a sheet and its entered into a computer program and if he called [his local Alzheimer’s TN Chapter], they could immediately meet him where he was.
Individuals involved	Self-efficacy	Facilitators have wealth of caregiver support experience Strong organizational resources	Professional experience Personal experience Community knowledge Community connection	For me personally, I have a long background in Alzheimer’s dementia care, and a background as a clinician therapist, and I facilitated many support groups in-person. Great thing about Alzheimer’s Tennessee as an umbrella organization is they have knowledge, information and connections in the community. If we don’t know it, we can find that information
Process	Executing	Lack of initial planning of long-term virtual services Initial conference calls gradually lost participation	Rapid implementation Interactive Zoom calls Conference call difficulties	Back then, we thought “Oh, we’ll be back together in the summer.” We’re starting to think that these folks, because they have suppressed immune systems, they will be the last that will want to mingle together. We didn’t do Zoom until August, and conference call numbers dwindled to 0–2 people per call. As soon as we tried Zoom, we get at least 10 people each week. Some of our folks who call in, they may have some technical issues like with screens freezing and maybe there’s a delay in the phone calls, but that’s just technology and we can’t control it. What we’ve learned to do, is we’re very flexible, of course you have to be anyway. Since it is informal, we can laugh together, we can cry together if you want to do that too, it’s part of the norm when you’re doing this.
Intervention	Relative priority	Would rather have in-person support group meetings Hybrid model likely in the future	Non-verbal communication barriers Technological barriers Effective support tool	While we do love and appreciate virtual support groups, I will say in-person is always better. It’s better because you can actually see someone. Like when you’re doing a virtual support group, as you probably know, you have a gallery of course and we all can see each other but we can’t touch each other and we can see some body language because you know nonverbal’s about 80% of our communication anyway. We want to really be able to support people and give them another tool to provide care and support for themselves. It’s all worthwhile so that’s why I hope we keep some of these continued past the deadline.
	Design quality and packaging	Ability to see and hear participants was beneficial	Participant-driven Non-verbal body language	There’s some structure, but it’s a loose, flexible structure… I try to be flexible because I’m aware we have caregivers along all sections of a journey, and so if we have a new person… I gave [them] longer to speak, and other people chimed in, which helped them in their journey because there’s something powerful about helping somebody else.
	Reflecting and evaluating	Piloting the program would have been helpful	Troubleshooting Learning with caregivers	It would have been nice to play around with the virtual platform so I could best talk people through any problems they had getting on, but we were learning together.

## Data Availability

The data contained in this study are available on request from the corresponding author.
